# The attenuation of retinal nerve fiber layer thickness and cognitive deterioration

**DOI:** 10.3389/fncel.2013.00142

**Published:** 2013-09-19

**Authors:** Yuan Shen, Zhongyong Shi, Renbao Jia, Yikang Zhu, Yan Cheng, Wei Feng, Chunbo Li

**Affiliations:** ^1^Department of Psychiatry, Tenth People’s Hospital, Tongji UniversityShanghai, People’s Republic of China; ^2^Department of Psychiatry, Tongji Hospital, Tongji UniversityShanghai, People’s Republic of China; ^3^School of Medicine, Tongji UniversityShanghai, People’s Republic of China; ^4^Department of Biological Psychiatry, Shanghai Mental Health Center, School of Medicine, Shanghai Jiao Tong UniversityShanghai, People’s Republic of China

**Keywords:** biomarker, Alzheimer’s disease, mild cognitive impairment, dementia, retinal nerve fiber layer thickness; cognition

## Abstract

Thinner retinal nerve fiber layer (RNFL) has been reported in Alzheimer’s disease (AD) patient. However, whether changes in RNFL thickness can predict the cognitive deterioration remains unknown. We therefore set out a prospective clinical investigation to determine the potential association between the attenuation of RNFL thickness and the deterioration of cognitive function over a period of 25 months. We assessed cognitive function using the Repeatable Battery for the Assessment of Neuropsychological Status and measured RNFL thickness employing optical coherence tomography in 78 participants (mean age 72.31 ± 3.98 years, 52% men). The participants were categorized as stable participants whose cognitive status remained no change (*N* = 60) and converted participants whose cognitive status deteriorated (*N* = 18). We found that there was an association between the attenuation of superior quadrant RNFL thickness and the deterioration of cognitive function in the stable participants. In the converted participants, however, there was an inverse association between the reduction of inferior quadrant RNFL thickness and decline of cognitive functions [scores of list recall (*R* = -0.670, *P* = 0.002), adjusted (*R* = -0.493, *P* = 0.031)]. These data showed that less reduction in the inferior quadrant of RNFL thickness might indicate a higher risk for the patients to develop cognitive deterioration. These findings have established a system to embark a larger scale study to further test whether changes in RNFL thickness can serve as a biomarker of AD, and would lead to mechanistic studies to determine the cellular mechanisms of cognitive deterioration.

## INTRODUCTION

Alzheimer’s disease (AD), one of the greatest public health problems in the world, begins with a long asymptomatic period (pre-clinical stage of AD) when only its neuropathogenesis is progressing (Jack et al., 2010). Individuals with evidence of such neuropathogenesis, which could potentially be demonstrated by biomarkers, are at an increased risk to develop cognitive impairment and dementia (Price and Morris, 1999). The preclinical detection of such biomarkers would enable earlier and more effective interventions to those at high risk to develop AD (Sperling et al., 2011). Therefore, identifying AD biomarkers, especially the ones in the early stage of AD, is important (McKhann et al., 2011).

Thickness of retinal nerve fiber layer (RNFL) starts to decline approximately at age 20 and the reduction of RNFL thickness could be part of the pathology of neurodegenerative diseases including AD (Parikh et al., 2007). Reduction in RNFL thickness is therefore a potential early biomarker of AD (Ho et al., 2012). It has been reported that subjects with AD or mild cognitive impairment (MCI) may have thinner RNFL as compared to age-matched control subjects (Tsai et al., 1991; Hedges et al., 1996; Paquet et al., 2007; Kesler et al., 2011). However, whether the reduction of RNFL thickness over time can predict the progress of cognitive deterioration, remains to be investigated.

In this prospective clinical investigation, we assessed the potential association between the attenuation of RNFL thickness and the deterioration of cognitive function over a period of 25 months in two groups of participants: the participants whose diagnosis of cognitive status (e.g., normal cognition or MCI) remained unchanged (stable group, *N* = 60) and the participants whose diagnosis of cognitive status deteriorated (converted group, *N* = 18). We have hypothesized that the association of the attenuation in RNFL thickness and cognitive deterioration is different between the participants in the stable group and the participants in the converted group. We employed the repeatable battery for the assessment of neuropsychological status (RBANS) and optical coherence tomography (OCT) to measure the cognitive function and RNFL thickness, respectively, before and after a period of 25 months. Then we assessed the potential association between the attenuation in RNFL thickness and the deterioration in cognitive function in the participants of the stable and the converted group.

## METHODS

### PARTICIPANTS

This study was approved by the Human Research Ethics Board of affiliated Tongji Hospital of Tongji University in Shanghai, P. R. China [LL(H)-09-04]. All participants signed the written informed consent before being enrolled in the study.

The baseline assessment of cognitive function was conducted from October 2010 to November 2010 and the follow-up assessment of the cognitive function was conducted from November 2012 to December 2012 (a 25 months follow-up study). A total of 104 participants were screened and enrolled initially. All participants were older adults with normal functional capacity and lived independently at the time of screening. They were recruited from three communities in Shanghai, P.R. China via flyer distribution. The eligibility criteria at the time of baseline assessment included: (1) age 70 years or older; (2) 5 years or more of education; (3) Chinese as the first language. Participants were excluded if they had: (1) AD dementia or Parkinson’s disease dementia diagnosed according to DSM-IV (America Psychiatric Association, 2000); (2) history of mental disorders (e.g., depression and schizophrenia) diagnosed according to DSM-IV; (3) severe functional decline (e.g., unable to live independently) confirmed by relatives; (4) history of pre-existing cerebrovascular disorders; (5) known diseases affecting the eye or optic nerve, e.g., glaucoma or increased intraocular pressure (IOP), retinal detachment, retinal degeneration; and (6) hyperglycemia (fasting blood glucose ≥7.0 mmol/L). The participants were first screened for dementia by using the Chinese version of the MMSE (CMMSE; Katzman et al., 1988), Chinese version of Activities of the Daily Living Scale (ADL; Chen et al., 1995) and the Chinese version of the RBANS (Cheng et al., 2011) by three trained research assistants through face-to-face interviews. Then, psychiatrists made clinical evaluations of patients including history, physical examination, and checking basic laboratory results. According to Petersen’s definition (Petersen et al., 1999), participants were diagnosed with MCI if they had (1) self-reported memory problems for over 3 months, (2) normal function of daily living: the Chinese version of ADL ≤ 15 (Li et al., 2006), (3) normal general cognitive function but abnormal memory functioning evidenced by lower score of CMMSE than education-dependent cutoff points: 20/21 for those who had 6 years of education or less (elementary school), and 24/25 for those who had more than 6 years of education (middle or high school). Note this cutoff point was used in the similar studies in Chinese population (Li et al., 2006; Ma et al., 2008). The cutoff point of CMMSE was lower in China than in the United States due to the difference in education and culture (Zhang et al., 1990; Sahadevan et al., 2002; Zhou et al., 2006). Dementia was determined according to the DSM-IV criteria (Tzamalis et al., 2009). Participants were defined as “stable” if they changed neither from normal cognition to MCI nor MCI to dementia at the follow-up assessment. Participants were defined as “converted” if they changed from normal cognition to MCI or from MCI to dementia at the follow-up time. The diagnosis of MCI or dementia was made only if two psychiatrists confirmed the diagnosis.

### OPTICAL COHERENCE TOMOGRAPHY EVALUATION

OCT examination was conducted on the same day as the cognitive assessment. Repeat-scan protocol is proved to be a precise method for measuring RNFL thickness in normal eyes (Cheung et al., 2008). OCT (ZEISS Cirrus^TM^ HD-OCT 4000 OCT(2010 Carl Zeiss Meditec, Inc., Dublin, CA, USA) and IOP were performed in all participants as described by Cheung et al. (2008) with modification. The polarization and Z-offset, as determined from the OCT settings, were optimized to assure the best possible scanning quality before the scan was obtained. A default optic disk cube 200 × 200 protocol (software version 4.6) was used to determine the RNFL thickness. Layer-seeking algorithms found the RNFL inner (anterior) boundary and RNFL outer (posterior) boundary for the entire cube, except for the optic disk. A scan was saved only if the fundus image was sufficiently visible to distinguish the optic disk and the scanning circle and if there were no obvious movement artifacts with missing data at the acquired scan pattern. Images with eye movements during scans, poor centration, poor focus, low analysis confidence or signal strength less than 4/10 were excluded. RNFL thickness was measured three times per quadrant using repeat scan protocols and the average of the 12 values were used for each eye. The RNFL thickness (global, superior, inferior, nasal, and temporal quadrant) in each participant was the average from both eyes or from one eye if the data from the other eye were missing. Changes of RNFL thickness were defined by using the follow-up RNFL thickness minus the baseline RNFL thickness in the global and in each quadrant of RNFL.

### NEUROPSYCHOLOGICAL EVALUATION

The CMMSE was administered to assess general cognitive function as a screening instrument. The CMMSE is a translated version of the MMSE and has good validity and reliability in the Chinese population (Katzman et al., 1988). The Chinese version of the ADL (Chen et al., 1995) was administrated to evaluate the daily living function. It is composed of a Physical Self-Maintenance Scale (PSMS; six items) and an Instrumental Activities of Daily Living Scale (IADL; eight items). PSMS items include eating, bathing, dressing, grooming, transferring (e.g., moving from chair to bed and return), and using the toilet. IADL items include use of the telephone, shopping, preparing a hot meal, doing housework, taking medication, managing financial matters, getting to places beyond walking distances, and doing laundry. The Chinese version of RBANS (Form A) was used to evaluate the specific domains of cognitive function of the participants. The reliability and validity of RBANS in the Chinese elderly community has been validated using the American norm (Zhang et al., 2008). Scores were calculated according to the American norm and Chinese representative normal people and they were highly correlated to each other in all age groups (Lim et al., 2010; Yang et al., 2010). The test scores and the index scores, including the ones of immediate memory, visuospatial/constructional, language, attention, and delayed memory, were used for the data analysis. Changes of cognitive performance were calculated by using the follow-up scores minus the baseline scores of each test or domain. All neuropsychological assessments were conducted by trained research assistants according to the protocol.

### STATISTICAL ANALYSIS

Student’s *t*-test was used to compare demographic, cognitive, and retinal features between the participants in the stable versus converted group. Pearson’s correlation was performed to assess the correlation between the changes in RNFL thickness and the changes in cognitive function. Next, a linear regression model was used to adjust for confounders including age, gender, education, blood glucose, and blood cholesterol level, which were reported to influence RNFL thickness (Oshitari et al., 2009; Kardys et al., 2013). Scores of RBANS were treated as the outcome and modeled by RNFL thickness and these confounders in the regression. Analyses were performed using SPSS 17.0 for Windows, (SPSS Inc., Chicago, IL, USA), with *P *< 0.05 as the significance level.

## RESULTS

### DEMOGRAPHIC CHARACTERISTICS

**FIGURE 1:**
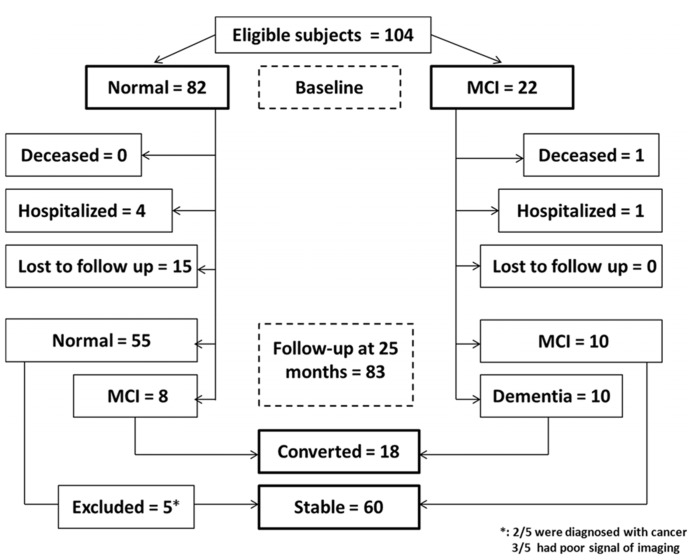
**Flow Diagram.** The diagram shows that 104 participants were initially screened for the studies and finally 78 participants were included in the data analysis.

There were 104 participants at the beginning (baseline), 82 were cognitively normal and 22 had MCI. Among the 82 cognitively normal participants at baseline, four withdrew from the studies for the treatment of respiratory infections (two) and bone fracture (two). Another fifteen participants were lost from follow-up due to “no more interest” (four), “unavailable” (one) or loss of contact information (10; see **Figure 1**). Among the 22 MCI patients, one died from a traffic accident and another one withdrew from the studies due to suffering from other illnesses. Thus, a total of 83 participants were assessed at 25 months after enrollment.

Three of the 83 participants were excluded because they were not able to provide a clear image of RNFL in both eyes by OCT measurement, two of them were diagnosed with cancer before the final assessment (see **Figure 1**). Therefore, 78 participants were included in the final data analysis, among them, 58 had normal cognition at baseline and 20 had MCI at baseline. The mean age of participants at the time of baseline was 72.31 ± 3.98 (mean ± SD) years, 52.0% participants were male.

At the time of 25 months after the beginning of the studies, 50 of the 58 participants who were cognitively normal at the baseline remained cognitively normal and 8 of the 58 participants converted to MCI. Among 20 participants who had MCI at the baseline, 10 of them stayed as MCI and 10 of them converted to mild (9) or moderate (one) dementia. Therefore, 18 participants who converted from normal cognition to MCI or from MCI to dementia were defined as “converted.” The other 60 participants who did not convert from normal cognition to MCI nor from MCI to dementia were defined as “stable” (**Table 1**). Student’s *t*-test showed that the converted participants had lower education years than the stable participants: 6.95 ± 2.23 (mean ± SD) years versus 10.23 ± 3.65 (mean ± SD) years (*P *< 0.001). As expected, the converted participants had lower MMSE scores than the stable participants (*P *< 0.001; **Table 1**). There were no differences in age, sex ratio, blood pressure, and level of blood cholesterol and glucose between the stable and converted participants (**Table 1**).

**Table 1:** Demographics characteristics for participants assessed at 25 months.

	Stable	Converted	*P*
Number	60	18	
Age (years) (SD)	74.1 (3.7)	75.3 (4.1)	0.27
Male sex (No.) (%)	26 (43.3%)	8 (44.4%)	0.821
Education (years) (SD)	10.23 (3.65)	6.95 (2.23)	<0.001
SBP (mmHg) (SD)	132.6 (15.5)	135.1 (9.3)	0.54
DBP (mmHg) (SD)	77.1 (7.7)	73.8 (7.3)	0.13
Cholesterol (mmol/L) (SD)	1.4 (0.5)	1.7 (0.7)	0.19
Blood glucose (mmol/L) (SD)	5.5 (1.3)	5.8 (1.7)	0.41
MMSE (score) (SD)	27.7 (1.7)	24.6 (3.1)	<0.001

*P value refers to differences between groups of stable and converted in age, sex, education, MMSE, SBP, DBP, cholesterol, and blood glucose. Education refers to self-reported years of education. The difference of sex was determined by Chi-square test, the difference in other variables was determined by the student’s t-test. MMSE, mini-mental state examination; SBP, systolic blood pressure; DBP, diastolic blood pressure.*

### ASSOCIATION BETWEEN THE ATTENUATION OF RNFL THICKNESS AND DETERIORATION OF COGNITIVE FUNCTION IN THE STABLE PARTICIPANTS

In the stable participants, Pearson’s correlation analysis showed that the reduction of RNFL thickness in the superior quadrant was positively associated with the reduction in score of list recall (*R* = 0.279, *P* = 0.031) and delayed memory (*R* = 0.328, *P* = 0.011), but not with other cognitive tests. Linear regression analyses were performed to evaluate the association between them. The Linear regression, after the adjustment of age, gender, education, blood glucose, and cholesterol level, showed that the reduction of RNFL thickness in the superior quadrant was still positively associated with a reduction of test score on the list recall (*R* = 0.294, *P* = 0.025) and index score of delayed memory (*R* = 0.335, *P* = 0.010; **Table 2**). These findings suggest that the attenuation of RNFL thickness was associated with the decline of cognitive function in stable participants. Specifically, more attenuation of RNFL in the superior quadrant may predict greater cognitive deterioration. Reduction in the inferior, nasal, temporal quadrant, and the global RNFL thickness was not associated with the reduction in the score of the cognitive test (data not shown).

**Table 2:** Association between reduction of RNFL thickness in the superior quadrant and cognitive deterioration in stable participants.

Cognitive function	Non-adjusted		Adjusted	
	*R*	*P*	*R*	*P*
List learning TS	0.076	0.562	0.048	0.734
List recall TS	0.279	0.031	0.294	0.025
Story recall TS	0.143	0.275	0.153	0.273
Immediate memory IS	0.089	0.532	0.085	0.542
Delayed memory IS	0.328	0.011	0.335	0.010

*The left panel of the table illustrates the results of the regression coefficients for the changes of RNFL thickness and cognitive function in a simple linear regression. The right panel of the table shows the regression coefficients in multiple linear regressions including adjustment of age, gender, education, blood glucose, and cholesterol levels. TS, test score; IS, index score.*

### INVERSE ASSOCIATION BETWEEN THE REDUCTION OF RNFL THICKNESS AND COGNITIVE FUNCTIONS IN THE CONVERTED PARTICIPANTS

Next, we asked whether the converted participants with a greater reduction of RNFL thickness also had a more significant decline of cognitive function. Pearson’s correlation analysis showed that the reduction of the thickness of inferior quadrant of RNFL was inversely associated with the reduction of test scores of list recall (*R* = -0.670, *P* = 0.002), story recall (*R *= -0.472, *P* = 0.048), the index scores of immediate memory (*R *= -0.555, *P* = 0.017) and delayed memory (*R* = -0.494, *P* = 0.037; **Table 3**). There was no association between the RNFL thickness in the superior, nasal and temporal quadrant with the test or index score of the cognitive function (data not shown). The liner regression analysis, with the adjustment of age, gender, education, blood glucose, and cholesterol level, showed that the reduction of RNFL thickness in the inferior quadrant was still inversely associated with a decrease in test scores of the list recall (*R* = -0.493, *P* = 0.031), story recall (*R* = -0.472, *P *= 0.048), and a reduction in the index score of delayed memory (*R* = -0.589, *P* = 0.033; **Table 3**). These findings indicate that the attenuation of RNFL thickness was inversely associated with the decline of cognitive function in converted participants. Specifically, less attenuation of RNFL in the inferior quadrant may predict greater cognitive deterioration. Reduction in superior, nasal, temporal quadrant, and the global RNFL thickness was not associated with the reduction in the score of the cognitive test (data not shown).

**Table 3:** Inverse association between reduction of RNFL thickness in the inferior quadrant and cognitive deterioration in converted participants.

Cognitive function	Non-adjusted		Adjusted	
	*R*	*P*	*R*	*P*
List learning TS	-0.307	0.216	-0.087	0.745
List recall TS	-0.670	0.002	-0.493	0.031
Story recall TS	-0.472	0.048	-0.472	0.048
Immediate memory IS	-0.555	0.017	-0.459	0.079
Delayed memory IS	-0.494	0.037	-0.589	0.033

*The left panel of the table illustrates the results of the regression coefficients for the changes of RNFL thickness and cognitive function in a simple linear regression. The right panel of the table shows the regression coefficients in multiple linear regressions including adjustment of age, gender, education, blood glucose, and cholesterol levels. TS, test score; IS, index score.*

## DISCUSSION

The RNFL thickness in healthy old adults is heterogeneous (Poinoosawmy et al., 1997; Parikh et al., 2007), therefore, it is important to assess the changes in RNFL thickness over time and to determine the potential association between the changes in RNFL thickness and the changes in cognitive function. We performed such studies over a 25 month-period in the participants who maintained their cognitive status and in the participants who had deterioration in their cognitive status.

We found that, whereas the more attenuation of RNFL thickness in the superior quadrant may predict greater cognitive deterioration in the participants who maintain stable cognitive function (neither converted from normal cognition to MCI nor from MCI to dementia), the less attenuation of RNFL thickness in the inferior quadrant may predict greater cognitive deterioration in the participants who have converted from normal cognition to MCI or from MCI to dementia. These findings suggest that patients who have less reduction in the thickness of the inferior quadrant of RNFL over the time may have a higher risk of developing MCI and dementia. Pending on further studies, these findings may indicate that the reduction of RNFL thickness could serve as a biomarker of MCI and dementia.

The findings that more attenuation of RNFL thickness in the superior quadrant may predict greater cognitive deterioration in the participants who maintain stable cognitive status (e.g., participants do not convert from normal cognitive function to MCI) suggest that the attenuation in the thickness of superior quadrant of RNFL may reflect a normal aging process, pending on further studies.

Previous studies (Kesler et al., 2011) have suggested that inferior quadrant of retinal nerve may be a more specific area than other quadrants of retinal nerve to reflect the retinal abnormity in early stage of AD, e.g., MCI. Consistently, our studies indicated that the changes in the inferior quadrant of RNFL thickness were more sensitive than thickness in other quadrants of RNFL in predicting the deterioration of cognitive status.

Impairment of delayed episodic memory is among the earliest signs of MCI patients who subsequently progress to AD (Schönknecht et al., 2005; Albert et al., 2011). In the present study, the attenuation of RNFL thickness was selectively associated with the decline in the delayed episodic memory but none of other cognitive domains, e.g., attention, language, or visuospatial construction, in the converted participants (data now shown). Collectively, we postulate that the attenuation of thickness of inferior RNFL could potentially be an early marker of cognitive decline in elderly people. Particularly, the attenuation of thickness in the inferior quadrant of RNFL over the time could be more sensitive to the cognitive deterioration than the changes in the global and other quadrants of RNFL.

The mechanisms underlying the inverse association between cognitive function and thickness of inferior quadrant RNFL in the participants who have deterioration of cognitive status remain unknown. Retinal nerve fiber projects to the calcarine fissure (occipital region) via lateral geniculate body and optic radiation (Mandala et al., 2012), which constitutes the periventricular white matter (Wilson and Steiner, 1986). White matter hyper-intensity (WMH) is commonly observed in AD patients, which is illustrated by increased intensities of white matter demonstrated by magnetic resonance imaging (MRI; Scheltens et al., 1993). Previous studies have reported that WMH are associated with an increased risk of dementia (Bocti et al., 2005) and rapid declines in global cognitive function (Ku et al., 2011). Two latest studies proved that periventricular WMH (PWMH) is inversely correlated with hippocampus volume as well as performance of episodic memory in AD patients (Kandiah et al., 2013), and was also inversely correlated with the cognitive deterioration in PD patients (Jang et al., 2013). Further studies, determining the association of RNFL thickness with WMH and other biomarkers of AD, e.g., hippocampus volume, CSF Aβ and/or Tau, are warranted to determine the underlying mechanisms of our clinical observation.

These findings would also lead to mechanistic studies to determine the cellular mechanisms of cognitive deterioration. The magnocellular pathway, which is associated with retinal nerve fiber, has its primary receptors in the retina (Alexander et al., 2001). Magnocellular pathway dysfunction has been identified in AD patients. Specifically, the magnocellular pathway in AD patients shows significant cell loss in the primary visual cortex (Hof and Morrison, 1990), reviewed in (Valenti, 2010). Collectively, we postulate that changes in retinal nerve fiber thickness may cause alterations in axon, which then affect synaptic function, leading to cognitive deterioration. The future studies, both in animals and in cultured neurons, are warranted to test this hypothesis.

The studies have several limitations. First, the drop-off rate at the time of the later assessment was relatively high (20.2%) and this may influence the interpretation of the data. However, we compared the demographic characteristics (in **Table 1**) of the participants who stayed for the study and the participants who dropped from the study, and we did not find significant differences in demographic features between the two populations. Second, the sample size of present study was relatively small. However, the current research serves as a pilot study to establish a system to further determine the potential value of RNFL thickness as a biomarker of AD and MCI in the future. Future studies with larger sample size and longer term observation should be performed.

In conclusion, our studies have demonstrated for the first time that longitudinal attenuation of RNFL is associated with cognitive decline. Specifically, the less reduction in the inferior quadrant of RNFL thickness would indicate a higher risk for the patients to develop MCI and dementia. These studies have established a system so that we can now embark on larger scale studies to further test the potential association between the attenuation of RNFL thickness and cognitive deterioration, which may ultimately lead to the development of employing RNFL thickness as a biomarker of AD. 

## Conflict of Interest Statement

The authors declare that the research was conducted in the absence of any commercial or financial relationships that could be construed as a potential conflict of interest.

## AUTHOR CONTRIBUTIONS

Study concept, design and funding obtain: Yuan Shen and Chunbo Li. Analysis and interpretation of data: Yuan Shen, Chunbo Li and Zhongyong Shi. Participant enrollment and cognitive evaluation: Zhongyong Shi, Renbao Jia, Yikang Zhu, Wei Feng and Yan Cheng. Drafting of the manuscript: Yuan Shen. Chunbo Li and Yuan Shen had full access to all of the data and the accuracy of the data analysis.
